# MAPK activity as a surrogate of acral melanoma immunogenicity?

**DOI:** 10.1111/jdv.20878

**Published:** 2025-09-26

**Authors:** Rastine Merat

**Affiliations:** ^1^ Dermato‐Oncology Unit, Division of Dermatology University Hospital of Geneva Geneva Switzerland

While acral melanoma, and particularly its dominant histological subtype, acral lentiginous melanoma (ALM), represents only a minor subset of melanomas in Caucasian populations, it is arguably the most universally occurring form of melanoma, affecting individuals regardless of skin phototype. Interestingly, and contrary to intuitive assumptions, the biological divergence observed in acral melanoma and more specifically in ALM, characterized by a distinct genomic trajectory,^1^ including delayed MAPK pathway dependence^2^ and an immunosuppressive tumour microenvironment,^3^ may also extend, at least in part, to other histological subtypes arising at acral sites, particularly the nodular subtype.

This possibility of an atypical, delayed MAPK activation pattern, in contrast to the canonical evolutionary trajectory of non‐acral melanomas, compels us to adopt a more nuanced framework for understanding acral and other non‐UV‐driven melanomas. Such a framework should move beyond the traditional dichotomy of *BRAFV600*‐mutant versus non‐mutant melanomas and instead evaluate MAPK pathway activity itself as a potential biological surrogate for tumour immunogenicity.

As an initial step towards this more refined stratification of acral melanoma, spatial transcriptomic technologies applied to a limited number of samples offer a valuable starting point. This approach can help determine whether, within a spatially heterogeneous tumour microenvironment, MAPK activity in malignant cells correlates with a specific immune infiltration profile. In the context of acral melanoma, such findings would be consistent with observations previously described in melanoma more broadly, where MAPK pathway activation has been associated with an immunosuppressive tumour microenvironment.^4^

This is precisely one of the key focuses of the study by Yang et al.,^5^ reported in this issue of *JEADV*. Spatial transcriptomic analysis was performed on a selected number of samples extracted from a larger cohort of acral melanoma patients to assess the immune landscape in relation to *BRAF* expression levels. The authors used transcriptomic profiling to analyse both the CD3‐positive lymphocytic microenvironment and the gene expression profiles of malignant melanocytes, stratified by *BRAF* expression level. Their findings indicate that, in acral melanomas, high *BRAF* expression is associated with an immunosuppressive microenvironment. In the ‘BRAF‐high group’, this was evident at the cellular level through neutrophil enrichment identified by cell‐type deconvolution and a reduced presence of activated natural killer cells, specifically revealed using the CIBERSORT algorithm. At the transcriptomic level, this was further supported by the downregulation, among others, of pathways related to antigen presentation.

Assuming, mainly based on copy number variation analysis including those from The Cancer Genome Atlas, and although this is clearly open to criticism, that *BRAF* mRNA levels reflect, to some extent, MAPK pathway activity and dependency, these results support the idea that, despite the distinct genetic landscape and evolutionary background of acral melanomas, some fundamental principles of melanoma biology may still apply. To strengthen and contextualize these findings, it would be valuable for the authors to disclose the histological subtypes of the tumours subjected to spatial transcriptomics. Specifically, identifying whether “BRAF‐high” tissues with suppressed immune signatures originate from superficial spreading melanoma (SSM), nodular melanoma or ALM would significantly enhance interpretability. Moreover, given that 16 ALM tumours in the study were identified as *BRAFV600E*‐mutated, such stratification could, in fact, clarify whether *BRAF*‐mutant ALMs behave more like their SSM counterparts biologically and immunologically.

We are confident that such an analysis will be performed in future works as the one carried out by Yang et al. Clarifying these distinctions is important for refining therapeutic approaches. More broadly, aligning omics‐based molecular profiles with classical histopathological subtypes will contribute to a more integrated understanding of melanoma biology, one that connects molecular findings with the extensive clinical and pathological knowledge that we have already accumulated.

## CONFLICT OF INTEREST STATEMENT

R.M. reports study funding paid to his institution from MSD, Philogen; honoraria for lectures, presentations or educational events from Regeneron, Meda pharma, Almirall; support for attending meetings and/or travel from Pierre Fabre pharma; participation on advisory board from Regeneron and is listed as inventor on a patent on the use of agents enhancing HuR/ELAV protein levels in the treatment of BRAF‐mutated cancers.

## Data Availability

Data sharing is not applicable to this article as no new data were created or analysed in this study.
